# PReS-FINAL-2052: Costs of biologics in juvenile idiopathic arthritis: past, present and future

**DOI:** 10.1186/1546-0096-11-S2-P65

**Published:** 2013-12-05

**Authors:** FH Prince, LW van Suijlekom-Smit

**Affiliations:** 1 Paediatrics/Paediatric Rheumatology, Sophia Children's Hospital ErasmusMC, Rotterdam, Netherlands; 2 Paediatrics, Amsterdam Medical Centre, Amsterdam, Netherlands

## Introduction

In the past decade biologics have changed the management of juvenile idiopathic arthritis (JIA). Many studies have evaluated the effectiveness and safety of the available biologics in JIA. Biological therapy used to be reserved for the severely ill JIA patients refractory to conventional therapy. Due to the treatment success of several biologics, more and more patients with JIA are now being treated with these drugs. In addition studies show that some patients may benefit from biologic therapy early in the disease course. However biologics are very expensive compared to conventional treatment. From a social-economic view the additional costs of new interventions should be weighted against their incremental health benefits compared to standard care.

## Objectives

We studied data on cost-effectiveness of biologics in JIA and evaluated the existing economic evaluations with implications for future research.

## Methods

We searched Medline, Embase, The Cochrane Library and The York Centre for Reviews and Dissemination database for relevant literature.

## Results

Current data on costs of biologics in JIA are scarce; five studies describe the costs of this treatment in JIA. Only three of these studies compare the costs with the treatment effect. These studies show that biologics are more costly than other rheumatic drugs but other direct and indirect treatment costs are reduced due to the effectiveness of biologics. Considering the economic pressure on the healthcare system, cost-effectiveness of a treatment is import factor in clinical practice. Unfortunately data on long-term cost-effectiveness of biologics or treatment strategies involving biologics are lacking. Since JIA is a chronic disease emphasis should also be placed on how biologics effect long-term progression of disability and translate this in quality-adjust life-years (QALYs). Cost-utility models compare treatment costs to effectiveness in QALYs and will be of great interest to policymakers.

## Conclusion

Biologics are expensive compared to other anti-rheumatic drugs. After evaluating the possible economic models to study drug costs, we suggest that future research should provide health economic evidence from usual practice settings that evaluate the cost-effectiveness of specific clinical targets in individual patients. Next to studying cost-effectiveness of biologics in JIA, also long-term cost-utility should be evaluated. These measurements provide valuable information on the long-term effect of a treatment from a social-economic perspective.

## Disclosure of interest

F. Prince: None declared, L. van Suijlekom-Smit Grant/Research Support from: Dutch Board of Health Insurances, Dutch Arthritis Association, Pfizer, Abbott, Consultant for: Roche, Novartis.

